# Fidelity and acceptability of implementation strategies developed for adherence to a clinical pathway for screening, assessment and management of anxiety and depression in adults with cancer

**DOI:** 10.1186/s13690-024-01293-6

**Published:** 2024-05-06

**Authors:** Sharon He, Heather Shepherd, Phyllis Butow, Joanne Shaw, Marnie Harris, Mona Faris, Afaf Girgis, The ADAPT Program Group, Philip Beale, Phyllis Butow, Josephine Clayton, Jessica Cuddy, Fiona Davies, Haryana Dhillon, Mona Faris, Liesbeth Geerligs, Afaf Girgis, Peter Grimison, Thomas Hack, Marnie Harris, Sharon He, Brian Kelly, Patrick Kelly, Laura Kirsten, Toni Lindsay, Melanie Lovell, Tim Luckett, Lindy Masya, Michael Murphy, Jill Newby, Don Piro, Melanie Price, Nicole Rankin, Joanne Shaw, Tim Shaw, Heather Shepherd, Rosalie Viney, Jackie Yim, Nicole Rankin

**Affiliations:** 1 Psycho-Oncology Co-operative Research Group (PoCoG), School of Psychology, The University of Sydney, Sydney, NSW 2006 Australia; 2 Susan Wakil School of Nursing and Midwifery, Faculty of Medicine and Health, The University of Sydney, Sydney, NSW 2006 Australia; 3 Ingham Institute for Applied Medical Research, South Western Sydney Clinical School, University of New South Wales, Kensington, NSW Australia; 4 Melbourne School of Population and Global Health, The University of Melbourne, Melbourne, VIC 3052 Australia

**Keywords:** Implementation strategies, Implementation research, Cancer, Clinical pathway

## Abstract

**Background:**

Implementation strategies are crucial to facilitate implementation success. To prepare and support implementation of a clinical pathway for screening, assessment and management of anxiety and depression in cancer patients (the ADAPT CP), six broad categories of implementation strategies; (1) Awareness campaigns, (2) Champions, (3) Education, (4) Academic Detailing and Support, (5) Reporting, (6) Technological Support, were developed. The aim of this paper is to describe the fidelity and acceptability of six categories of implementation strategies and any subsequent changes/adaptations made to those strategies.

**Methods:**

The ADAPT CP was implemented in twelve cancer services in NSW, Australia, as part of a cluster randomised controlled trial of core versus enhanced implementation strategies. Fidelity to and any subsequent changes to the delivery of the planned six categories of implementation strategies were captured using the ADAPT contact log, which recorded the contacts made between the ADAPT research team and services, engagement meetings and monthly meetings. To explore acceptability and awareness/engagement with the implementation strategies, interviews with a purposively selected staff sample across both study arms were held prior to implementation (T0), six months into implementation (T1) and at the end of the 12-month implementation period (T2). Interviews were thematically analysed across the six categories of strategies.

**Results:**

Delivery of all six categories of implementation strategies as planned was moderated by service context and resources and staff engagement. As such, for some implementation strategies, subsequent changes or adaptations to the content, mode of delivery, frequency and duration such as abbreviated training sessions, were made to optimise fidelity to and engagement with the strategies. Most strategies were perceived to be acceptable by service staff. Use of strategies prior to implementation of the ADAPT CP such as the engagement meetings and training sessions, positively impacted on ownership and preparedness to implement the ADAPT CP. Furthermore, ongoing support such as provision of additional training or monthly meetings facilitated increased awareness and engagement with the ADAPT program.

**Conclusion:**

Flexibility in delivering implementation strategies, and ensuring staff engagement with, and acceptability of those strategies, can support implementation of interventions within healthcare settings.

**Trial registration:**

The ADAPT CRCT was registered prospectively with the ANZCTR on 22/3/2017. Trial ID ACTRN12617000411347. https://www.anzctr.org.au/Trial/Registration/TrialReview.aspx?id=372486&isReview=true

**Supplementary Information:**

The online version contains supplementary material available at 10.1186/s13690-024-01293-6.

**Table:** 

**Textbox 1. Contributions to the literature**
• Deliberate selection and delivery of relevant implementation strategies is important for implementation success. Clear documentation of the delivery of strategies as planned, and understanding of acceptability of strategies is crucial to informing appropriate selection and design of implementation strategies.
• Whilst we found that implementation strategies were delivered and acceptable, delivery of implementation strategies as planned varied, and subsequent adaptations or changes were made to ensure fit to local context and to optimise delivery and engagement with strategies.
• These findings contribute to the limited literature, including the acceptability and how they are adapted in real-world trials.

## Introduction

Successful implementation of health interventions requires the delivery of relevant implementation strategies, defined as the methods or techniques developed to facilitate adherence to, and adoption and sustainability of an intervention [1, 2]. Many strategies have been developed to address barriers to implementation of health interventions and increase engagement of staff with these interventions [3, 4]. Deliberate selection and delivery of relevant implementation strategies is important to ensure implementation success [5, 6]. However, fidelity and acceptability of implementation strategies are under-reported [7]. Lack of thorough documentation about the delivery of strategies may limit our understanding of implementation success and hinder refinement of future efforts [2, 8, 7].

We selected a range of strategies to support implementation of a clinical pathway for identification and management of anxiety and depression in adult cancer patients (the ADAPT CP) [9]. The ADAPT CP, described in detail elsewhere [9, 10], involves iterative screening, triage to one of five steps (based on severity of anxiety and/or depression), and referral to interventions appropriate to each step [11]. The ADAPT CP also provides recommendations on staff responsibilities, timing and type of intervention to address each severity step [11]. An online Portal was developed to operationalise the ADAPT CP [12].

The ADAPT CP was implemented across twelve oncology services in New South Wales (NSW), Australia as part of a cluster randomised controlled trial (CRCT) [9]. The ADAPT CRCT aimed to evaluate two “doses” of implementation support (core versus enhanced) to facilitate adherence to the ADAPT CP [9]. The ADAPT CRCT Working Group, comprised of experts in the fields of psycho-oncology, oncology, implementation science and consumers, developed the suite of implementation strategies, informed by a systematic review of barriers to implementation [13], a qualitative analysis of barriers to implementation of the ADAPT CP [14] and local implementation practices.

Prior to implementation of the ADAPT CRCT, our team defined measures of implementation success according to Proctor et al’s [15] Implementation Outcomes framework. Fidelity was defined as “*the degree to which each service received the implementation strategies as planned within their randomization*” [16]. Acceptability of the implementation strategies was defined as “*cancer staff perceptions of the implementation strategies as agreeable, palatable or satisfactory*” [16].

This paper describes fidelity and acceptability of the six categories of implementation strategies. Where applicable, we also identified reasons for differences in the extent to which services engaged with the strategies as planned and any subsequent changes/adaptation made to the strategies.

## Method

### Study context

The ADAPT CRCT study procedure is reported in full elsewhere [9]. Briefly, services were randomised to two levels of implementation support (core versus enhanced). Prior to implementation, each service participated in 6-8 *Engagement Meetings* at which one or more local champions were identified and a multidisciplinary lead team was formed to tailor the ADAPT CP to local service resources and requirements [17]. Services then progressed to the “Go-Live” stage to implement the ADAPT CP for 12-months. See Additional file 1 for Consort Flow Diagram.

The study was approved by the Sydney local Health District Human Research Ethics Committee, Protocol X16-0378 HREC/16/RPAH/522.

### Implementation strategies

Six categories of implementation strategies were designed that aimed to prepare services for implementation and provide support throughout the 12-month implementation period: (1) Awareness campaigns, (2) Champions, (3) Education, (4) Academic Detailing and Support, (5) Reporting, and (6) Technological Support (Table 1).

**Table 1:** Description of core and enhanced implementation strategies^a^

**Implementation Strategy**	**Description**	**Core and Enhanced or Enhanced Only**	**Flexibility**
1.Awareness Campaigns			
1.1 Roadshows	One or more ADAPT Roadshows were delivered to all participating cancer services, ideally 8 weeks prior to CRCT implementation. The purpose of Roadshows was to inform staff about the ADAPT CP, outline associated resources for staff and patients, and in doing so raise staff awareness of and engagement with the program. Roadshows also provided an opportunity for non-lead team members to meet the ADAPT team and ask any questions.	Core and Enhanced	Flexible (tailored content and scheduling)
1.2 Poster Campaigns The poster campaign strategy aimed to raise staff awareness of ADAPT.			
1.2.1 “ADAPT Is Coming”	Intended display was four weeks prior to CRCT implementation/Go-Live, with removal at go-live.	Core and Enhanced	Flexible (tailored content)
1.2.2 “ADAPT Support”	Intended display was at Go-Live (first day of CRCT implementation), with removal at the end of the 12-month CRCT implementation period.	Core and Enhanced	
1.2.3 “ADAPT is Live”	Intended display was at Go-Live for enhanced services only, with removal one month later.	Enhanced Only	
1.2.4 “ADAPT Support” Refresh	An ADAPT Team member visited enhanced services at four and eight-months into CRCT implementation to check that the “ADAPT Support” poster was still hanging and, if not, re-hang them.	Enhanced only	
1.3 Go-Live Correspondence emails	A Go-Live email (outlining essential information about the ADAPT CP and Portal, the date of CRCT implementation and what staff needed to do in preparation for this) and a distribution list comprising staff names and email addresses was sent to the ADAPT Champion two weeks prior to Go-Live, with the intention that the Champion would circulate this to identified staff one week prior to Go-Live.	Core and Enhanced	Flexible (tailored content)
1.4 Newsletter	It was intended that five newsletters, at 9, 18, 26, 39 and 53 weeks into CRCT implementation, would be sent to identified staff at enhanced services. Newsletter content was tailored to each service, and contained information about service progress (registration, screening and referral numbers, and patient activity in the ADAPT Portal), reminders about the ADAPT CP and Portal, and links to resources. Newsletter content was an item on Monthly Meeting agendas, and staff were encouraged to contribute topics and/or content to be included. It was intended that some newsletters would contain a profile (photo and description) of staff involved in CP implementation, and updates from the local ADAPT Champion which service staff would contribute.	Enhanced only	Flexible (tailored content)
2.Champions	A local champion and portal site administrator (portal champion) were nominated at all participating cancer services, ideally during the first Engagement Meeting. The role of the local ADAPT Champion was to facilitate multidisciplinary support and involvement in planning and delivering the Clinical Pathway, to act as a conduit for communication between the ADAPT team and the local cancer service, and to promote the ADAPT Program to staff at their service and encourage engagement. The identification of at least one local Champion during the site recruitment phase was a prerequisite of trial participation. The Portal Site Administrator received more intensive Portal training (Super User Training) and had higher level access to Portal functions including staff registration and Portal configuration.	Core and Enhanced	Fixed
3. Education			
3.1 Clinical Pathway Overview Training	Clinical Pathway Overview Training aimed to highlight the importance of routine screening for anxiety and depression and increase staff knowledge of the ADAPT CP and stepped care model. Intended delivery was four weeks prior to Go-Live.	Core and Enhanced	Flexible (tailoring to available scheduling)
3.2 Portal Overview Training	The Portal Overview training aimed to give staff an understanding of the ADAPT Portal and its functionalities. Intended delivery was two weeks prior to Go-Live, and prior to the tailored Portal User Training.		
3.3 Portal User Training	Portal User Training was delivered to staff who would be using the ADAPT Portal, during Go-Live. Training was intended to be delivered one-on-one or in small groups, so it could be tailored to the tasks and needs of individual users and enable staff to ask questions.		
3.4 Super User Training	Super User Training was offered to the portal site administrator at each participating cancer service. This was more in-depth than the Portal User Training and covered additional Portal functionalities only accessible to the portal site administrator.		
4.Academic Detailing and Support			
4.1 Written Report on Interview Data	Interviews were conducted with purposively selected staff just prior to ADAPT CP implementation (T0) and again at 6 and 12 months into the 12-month supported implementation phase (T1 and T2, respectively). Following these, a report summarising the interview findings about staff knowledge of and beliefs regarding the ADAPT CP, workplace context, support to implement the ADAPT CP and barriers and facilitators to delivering the implementation strategies and ADAPT CP was compiled and sent to the local Champion at all participating services. Champions were encouraged to share this with staff involved in CP implementation, and enhanced services had the opportunity to discuss these reports with the ADAPT Team.	Core and Enhanced	Fixed
4.2 Engagement Meetings	It was intended that a series of 7-8 structured engagement meetings, ideally spaced two weeks apart, would be delivered to lead team members at all participating cancer services prior to implementation of the ADAPT CP. The aim of the Engagement Meetings was to tailor the ADAPT CP to service resources and requirements and increase ownership.	Core and Enhanced	Flexible (tailoring of ADAPT CP)
4.3 Monthly Meetings	It was intended that lead teams at enhanced services would meet with the ADAPT research team monthly throughout the 12-month supported implementation period to review service progress (via portal activity reports), identify any emerging implementation difficulties and brainstorm solutions. Meetings also provided the opportunity for staff to discuss portal functionality, ongoing training requirements, staff changes and CP sustainability. The meetings were scheduled for 30 minutes.	Enhanced only	Fixed
5.Reporting	A monthly report, detailing service progress (in terms of the number of patients registered, screened, triaged and referred) and recommendations from the ADAPT Team, was emailed each month to lead team members at all services.	Core and Enhanced	Fixed
	These reports were also reviewed during enhanced services’ monthly meetings.	Enhanced only	Fixed Fixed
6. Technical Support	IT support for the ADAPT Portal. Service staff could contact the ADAPT Support team prior to Go-Live (during the site engagement phase) with comments, queries with signing up or accessing the ADAPT Portal. Service staff and patients could contact the ADAPT support team throughout the 12-month implementation period with any issues, queries, comments related to using the ADAPT Portal.	Core and Enhanced	

^a^Table adapted and reproduced with permission (Shepherd et al. 2019, The elusive search for success: defining and measuring implementation outcomes in a real-world hospital trial, *Frontiers in Public Health*) [16]

Core strategies were consistent with usual practice for implementation of new guidelines or clinical pathways in the Australian context [18, 19] and delivered to both study arms. Strategies recommended depend on the perceived barriers and typically involve education materials and training to increase knowledge or understanding of the intervention being implemented, endorsing clinical champions to encourage other clinicians to use guidelines and promotion or communication campaigns such as posters to increase awareness [19]**.** Services randomised to the enhanced strategy arm received additional strategies and more active and sustained engagement with the ADAPT team throughout the 12-month implementation period: specifically, additional awareness campaigns and monthly face-to-face Lead Team meetings with the ADAPT Research team to discuss monthly reports and progress and identify and resolve any emerging issues. These ‘enhanced’ strategies were underpinned by Weiner’s (2009) [20] theory of organizational readiness for change, where we provided opportunities for the ADAPT team to facilitate services’ focus on what was working well and what wasn’t and to make adaptations and offer assistance where needed*.* A degree of flexibility and tailoring of implementation strategy delivery was permitted, to fit service scheduling requirements and enable content of awareness campaigns, meetings and training to be tailored to service or staff needs [16].

### Data collection: implementation strategy fidelity and engagement/adaptations

*Engagement Meetings* were recorded to capture discussion, decisions around tailoring of the ADAPT CP, and planned delivery of the implementation strategies.

An *ADAPT Contact Log* captured contacts made between services and the ADAPT team, including those related to scheduling and delivery of implementation strategies. Staff attendance at meetings where implementation strategies were delivered was noted, as were discussions about scheduling and delivery of the strategies. Ad hoc contacts related to the implementation strategies (e.g., modification of posters, requests for additional training) were also recorded by the research team.

*Monthly Meetings* held through the 12-month implementation period for sites randomised to the enhanced strategy arm were recorded, capturing discussions, decisions and resolution of any emerging issues with delivery of implementation strategies.

The number of newsletters sent to staff, opened and number of clicks on the newsletter links in the enhanced strategy arm services were recorded in the *Mailchimp* email platform.

### Data collection: implementation strategy acceptability and engagement

*Staff Interviews*: A subset of staff from each service, purposively selected to include members of the lead team, non-lead team and multi-disciplinary representation, to ensure representation of views of staff with varying degrees of interaction with ADAPT and from both study arms, were invited to participate in a semi-structured telephone interview at three time points: prior to ADAPT CP implementation (T0), 6-months into implementation (T1) and at the end of the 12-month implementation period (T2). An email invitation was sent to the identified service staff. The same recruitment method was used across all services, regardless of trial arm. The interview guide was developed to explore, amongst other issues, acceptability and awareness/engagement with the implementation strategies that supported ADAPT CP implementation. Interviews were conducted by three female trained qualitative researchers who had no direct involvement with ADAPT CP implementation. Interviews were audio-recorded and transcribed verbatim.

### Data analysis

Fidelity and adaptation/engagement data: Dates (e.g., date of delivering or displaying the awareness campaigns), timing (e.g., length of training sessions) and attendances (e.g., who attended) which were extracted from the ADAPT contact log and descriptively summarised in Microsoft Excel. Data related to implementation strategies from the engagement meetings, ADAPT contact log and monthly meetings were extracted and content analysed to identify subsequent changes/adaptations made to the strategies and reasons for differences between implementation strategies planned and delivered.

Acceptability and awareness/engagement data: Staff interviews across the three timepoints were thematically analysed [21] using NVivo12 software [22]. Two researchers (MH, SH) initially coded six transcripts from each timepoint to develop a draft coding framework, which was discussed with a third researcher (PB) and refined. All interviews were then independently coded line-by-line, with differences resolved through consensus. Similarities and differences in coding were examined to develop initial themes and reviewed to identify higher order themes. Themes and quotes were mapped to randomisation arm and timepoint, to enable any differences according to randomisation and implementation stage to be identified, as per the Framework Analysis method [23].

To explore the concepts of fidelity further, we identified the conceptual framework for implementation fidelity by Carroll et al., (2007) [24]. Carroll et al., (2007)’s conceptual framework of implementation fidelity, depicts four main components of fidelity (details of the content, coverage, frequency and duration) and four domains that act as moderators to fidelity (complexity of intervention, implementation strategies, quality of delivery and participant responsiveness) [24]. This framework was used in our discussion to illustrate the complexity of health services implementation.

## Results

All six implementation strategies were delivered to the twelve services. However, the extent to which services received the relevant strategies as planned differed within core and enhanced services. Adaptations to strategies were made to facilitate delivery of implementation strategies. Two hundred and fifty-three interviews were conducted with 122 different staff members over three timepoints at T0 (*n*=88), T1, (*n*= 89) and T2 (*n*=76). See Table 2 for interview participant characteristics. There were 167 staff interviews analysed in the enhanced arm and 86 staff interviews in the core arm. The acceptance and attrition rates for staff who participated in interviews have been described elsewhere [25]. In brief, the response rates across all three time points were 64% (70% at T0, 66% at T1, and 57% at T2). There were 87 staff (71%) who participated in multiple interviews. Staff perceptions of the strategies generally remained stable over time and between randomisation arm, however, any changes or differences were noted. Quotes are identified by study arm: enhanced (E) versus core (C); profession, oncology service (S1-12), personal ID and time of assessment (T0-2). Additional quotes are provided in Additional file 2.

**Table 2:** Staff interview participants: demographic and professional characteristics^a^

	**T0 (*n*=88)**		**T1 (*n*=89)**		**T2 (*n*=76)**	
	**n**	**%**	**n**	**%**	**n**	**%**
Age Range (in years)						
18-25	2	2.3	2	2.2	3	3.9
26-50	61	69.3	67	75.3	48	63.2
51-75	23	26.1	16	18.0	22	28.9
Missing	2	2.3	4	4.5	3	3.9
Gender						
Female	75	85.2	73	82.0	66	86.8
Male	13	14.8	16	18.0	10	13.2
Role^b^						
Nursing Staff	33	37.5	34	38.2	26	34.2
Medical Staff	12	13.6	13	14.6	8	10.5
Allied Health and Clinical Trials Staff	6	6.8	4	4.5	8	10.5
Administrative, technical support and non-clinical managers	15	17.0	12	13.5	13	17.1
Psycho-social Staff	22	25.0	26	29.2	21	27.6
Employment Status						
Full-time	57	64.8	58	65.2	49	64.5
Part-time	27	30.7	26	29.2	24	31.6
Part-time, independent contractor	2	2.3	0	0.0	0	0.0
Full-time, independent contractor	0	0.0	1	1.1	0	0.0
Missing	2	2.3	4	4.5	3	3.9
Language spoken at home						
English	77	87.5	74	83.1	65	85.5
Other^c^	9	10.2	11	12.4	8	10.6
Missing	2	2.3	4	4.5	3	3.9
Country of birth						
Australia	62	70.5	58	65.2	52	68.4
Other^d^	24	27.2	27	30.3	21	27.7
Missing	2	2.3	4	4.5	3	3.9
Aboriginal or Torres Strait Islander						
No	85	96.6	84	94.4	73	96.1
Yes, Aboriginal	1	1.1	1	1.1	0	0.0
Missing	2	2.3	4	4.5	3	3.9

^a^Table reproduced with permission (Butow et al., 2021, Acceptability and appropriateness of a clinical pathway for managing anxiety and depression in cancer patients: a mixed methods study of staff perspectives. BMC Health Services Research) [25]

^b^Roles included in the categories:

Nursing Staff: Nurse- RN/AIN, CNS, CNE Care Coordinator, CNC, NUM, Nurse Practitioner

Medical Staff: Oncologist, Haematologist, Psychiatrist, Registrar, Medical oncology Fellow

Allied Health & Clinical Trials Staff: Speech pathologist, Clinical Trials,

Admin, technical support & non-clinical managers: Admin, IT staff, Volunteer, Clinical Support Officer, Management, Program Coordinator, Practice Manager

Psychosocial staff: Psychologist, Psychologist Intern, Social Worker, Counsellor

^c^Other languages spoken at home: Cantonese, Indonesian, Malayam, Mandarin, Portuguese, Spanish, Tagalog

^d^Other countries of birth: Brazil, Canada, China, Hong Kong, India, Indonesia, Kenya, New Zealand, Peru, Philippines, South Africa, Sri Lanka, UK

### Fidelity and adaptations to awareness campaigns

#### Roadshows

At least one in-person Roadshow was delivered to each service to inform staff about the ADAPT CP (*Mean*=3 per service, range 1-5) to raise awareness and increase engagement with the ADAPT CRCT. Adaptations to the number, duration and timing of Roadshows were made based on availability of staff and space within cancer services. Roadshows were held later than the intended eight weeks prior to implementation, due to: non-availability of forums/meetings till later (*n*=2), intended meeting falling on a holiday (*n*=1); or compressing the whole engagement process to enable Go-Live before year-end (*n*=1). In total, 271 staff across the 12 services attended a Roadshow (*Mean*=23 per service) (Table 3).

**Table 3:** Roadshow delivery and attendance

**SID**	**Forum where Roadshow was delivered**	**Delivery**		**Attendance**
	**Existing meeting or additional meeting time?**	**Number of Roadshows held (n)**	**Total Duration (mins)**	**Total Attendances (n)**
1	Existing	1	30	15
2	Additional	4	120	15
3	Existing	1	30	10
4	Existing	4	100	29
5	Additional	4	120	15
6	Existing	5	60	48
7	Existing	2	50	16
8	Existing	1	30	23
9	Existing	1	20	7
10	Existing	4	90	43
11	Existing	3	55	24
12	Existing	3	82	26
**Total**		**33** (100%)	**787** (13hr 7m)	**271**
**Average**		**2.75**	**66** (1hr 6m) per service (24 m) per Roadshow	**22.58 per service**

For many participants, the Roadshow was their first exposure to the ADAPT CP; those who attended had positive perceptions of this strategy, feeling it gave staff a *“better understanding of what [the ADAPT CP]’s actually all about”* (E_NURS_S12P02T0).

Some participants suggested holding the Roadshow or a refresher session closer to Go-Live, or holding more Roadshows to maximise staff attendance and ensure information was retained.

### Reminder strategies:

#### Posters

All services received two sets of posters, the first (ADAPT Is Coming) displayed prior to go-live, the second (ADAPT Support information) displayed at Go-live (on the first day of implementation). Enhanced strategy arm services received an additional ADAPT is LIVE poster at Go-live for one month, and two refresh/replacement of the ADAPT Support poster at 4 and 8 months into implementation. Of the 48 posters displayed across all services, the majority (*n*=34) were displayed as planned; some were displayed earlier or delayed. Reasons for delay included governance or staff approval, requests for additional information on the poster, the Champion forgetting, and a decision to delay display until the ADAPT Team site visit.

Further adaptations to the poster strategy were noted in some services. Six services from the enhanced study arm requested additional patient-facing posters, or changes to the posters to fit local context (i.e., e-posters for display on electronic poster boards). Two services requested further communication emails to staff or patients to increase awareness.

#### Newsletters

Five e-newsletters, tailored to each of the eight enhanced strategy arm services, were sent to service staff across the implementation period, containing information on service progress, and reminders about the ADAPT CP and Portal. Most newsletters (*n*=37/40) were emailed to service staff within one week of their intended delivery date. Service staff (typically the Champion) were asked to review and approve newsletters prior to dissemination; delays or failure to respond meant some newsletters were delayed.

**Fig. 1:**
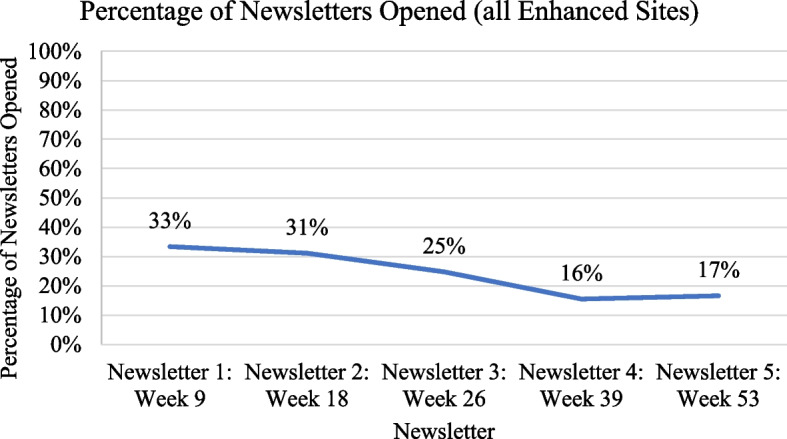
Percentage of newsletters opened over time across all enhanced strategy arm services

Across the 1036 newsletters sent, only 239 (24%) of newsletters received were opened. Across all services, 48% of staff opened at least one newsletter. In addition to written content, newsletters contained on average one link (range 0-2) to additional information, including online health professional training. However, clicks on newsletter links were very low: 7 clicks across all newsletters received (0.6% of maximum possible clicks). Services were encouraged to tailor newsletter content to their service; there was generally low engagement with this tailoring. See Fig. 1.

#### Go-live email correspondence (See Table 1, 1.3)

The ADAPT team sent a planned email, the “Go-Live Email Correspondence” which contained essential information about the ADAPT CP and Portal, the date of CRCT implementation and what staff needed to do in preparation for this, for site Champions to disseminate to staff, as planned in the eight core services.

Some participants reported having seen ADAPT posters and email correspondence, whilst others had not. Whilst some commented that these strategies acted as *“a constant reminder that… the program is there”* (NURS_S03P03T0) and helped to increase awareness particularly amongst non-lead team staff, others felt these were easily overlooked and thus not effective.

Participants commented that briefly mentioning ADAPT during existing meetings may be more effective to raise awareness amongst wider service staff. Overall, participants commented that the combined awareness strategies (Roadshows, posters, emails, newsletters) helped to increase wider service staff awareness prior to Go-Live.

*“with the posters and the emails, and the face-to-face sessions, it was really… capturing everybody’s attention… so when it went live… everybody was on board with it… we…registered lots of patients in the first few weeks which was really good.”* (E_AH_S06P03T2)

### Fidelity to champion correspondence

Champions at each service were sent six Portal “Tips and Tricks” emails, each containing a “Portal Tip” to be circulated by Champions to registered ADAPT Portal Users at 4, 8, 12, 16, 28 and 40 weeks into implementation. Most were disseminated as planned (62/72), the remaining were sent earlier (3/72) or later (7/72). We were unable to capture how many Portal Users received the “Portal Tip” email.

Most participants were aware who was nominated as the ADAPT champion at their service, believing that Champions played a critical role in driving service change, and in relation to ADAPT. More generally, these champions increased awareness and engagement of service staff including senior management and ensured responsibility for implementation was not diffused.

*“having key players… who check in and… rally a team… can be dependent if any issues come up… and just having someone who has relationships with the key players who can move things forward, I think that’s probably pretty critical.”* (E_PSYCH_S09P03T0)

Participants saw the ADAPT champion as a ‘go-to’ person who could train and upskill staff, communicate necessary information about the ADAPT CP and Portal, answer questions and troubleshoot issues as they arose. This eased the perceived burden of implementation, as one participant said:

*“I think yeah, the champions were key in that finding solutions to the implementation and being committed to doing it. And making sure everybody else who needed to do something, did their thing*.” (C_MED_S01P09T2)

#### Attributes of a successful champion

Participants commented that the champion’s role, authority and workload capacity could impact the success of this strategy.

#### Role

Clinical staff with regular patient contact were seen as well-placed to champion, tailor and enact the CP. They had well-established collegial relationships with staff implementing the CP, making them approachable if staff required assistance.

*“[champion]’s got a lot of clinical experience and especially in psycho-oncology…she’s very good at seeing how systems work and what the patients need clinically and trying to modify or trying to make ADAPT fit with the care, with the needs of the service so… I think she’d be good.”* (C_NURS_S11P04T0)

Having a champion who held a clinical role also meant this role could continue beyond the 12-month implementation period. Where the champion role was taken on by, for example, a clinical trials staff member there were concerns this role would not continue beyond the implementation period.

#### Authority

Participants commented on the importance of service change being championed or supported by senior management staff. At one service participants commented that champions *“lacked the power”* to secure staff engagement, which made this strategy less effective.

#### Workload Capacity

Participants acknowledged that championing ADAPT would require additional time and energy on top of the Champion’s existing clinical load. Hence the success of this strategy depended upon the champion’s ability to dedicate time to this role. For example, where funding was secured for a dedicated part-time role, the ADAPT CP implementation could be successfully prioritised.

*“I think that was the key because I think having someone dedicated to that role, means actually having it done. Whereas I think… if they get that added to their role as part of their current role… it can be quite difficult to add that to their workload.”* (C_NURS_S12P01T2)

On the flip-side, however, staff in the same service commented that having a designated champion limited wider service staff engagement.

*“having the champion also, to have it as her focus role is a positive thing, because I guess in that regard a number of us have stepped back thinking, well, that’s her role now.”* (C_AH_S12P06T0)

### Fidelity and changes to education

#### Clinical Pathway Overview Training

All services received CP Overview Training. Ideally, cancer service staff co-led this training with the ADAPT Program manager to increase ownership and staff engagement. Psychosocial staff co-led training at 9 services (Table 4). Delivery of training was delayed at some services. Changes to planned delivery of training was due to: service awaiting governance approval, delayed nursing staff approval of screening and scheduling issues.

**Table 4:** Attendance and duration of the clinical pathway, portal overview, portal user and super user training^1^

**SID**	**Clinical Pathway Training**			Portal Overview Training*			**Portal User and Super User Training**			**All Training Sessions**	
	**Sessions delivered (n)**	**Total duration (mins)**	**Total attendances (n)**	**Sessions delivered (n)**	**Total duration (mins)**	**Total attendances (n)**	**Sessions delivered (n)**	**Total duration (mins)**	**Total attendances (n)**	**Total training duration (mins)**	**Total training attendances (n)**
**1**	1	30	13	1	5	13	11	750	22	785	48
**2**	5	150	9	2	120	16	1	120	1^b^	390	26
**3**	1	30	10	1	90	6	10	410	23	530	39
**4**	1	25	16	1	25	16	7	400	17	450	49
**5**	3	45	11	3	45	14	15^a^	525	15	615	40
**6**	2	115	17	3	135	18	8	410	10	660	45
**7**	1	25	2	1	5	2	7	320	7	285	11
**8**	2	50	47	2	10	47	10	540	14	425	108
**9**	2	30	10	2	20	10	8	255	8	245	28
**10**	1	20	7	1	5	7	7	365	8	330	22
**11**	1	30	9	1	5	9	3	195	3	355	21
**12**	2	25	25	2	10	25	4	305	5	575	55
**Total**	**22 sessions**	**575 mins** (9h35m)	**176 attendances**	**20 sessions**	**475 mins** (7h55m)	**183 attendances**	**91**	**4595** (76h35m)	**133**	**5645** (94h5m)	**492**
**Average per service**	**1.83**	**48 mins**	**14.67**	**1.67**	**40 mins**	**15.25**	**7.58**	**383 mins** (6h23m)	**11.08**	**470** (7h50m)	**41.00**

^1^Total training attendances are not unique attendees, as some staff will have attended multiple trainings

^*^When CP and Portal Overview Trainings were combined, the same staff have been included in both CP Training and Portal Overview Training attendance numbers

^a^Included a short (15 min) training session by request

^b^No Portal user training, only 1 super user training reported

#### Portal overview training

Twenty Portal Overview Training sessions of on average 24 minutes (range 5-135 minutes) were held. Adaptations to this strategy included, providing abbreviated 5-minute, 2-3 slide Portal Overview presentation during their CP Overview Training upon site request instead of the training session (*n*=6 services) (Table 4). Reasons for this were due to difficulty finding sufficient time for staff to attend.

#### Portal user training

A total of 79 Portal User training sessions (average 43 minutes) were held (average 7 trainings per service, range 0-14 trainings). Most (*n*=59) were one-on-one, the rest (*n*=20) were in small groups (average group-size, *n*=3, range 2-9). A total of 121 Portal User Training attendances were recorded (*Mean*=10 attendances per service). All portal site administrators (*n*=12) at participating cancer services received the super user training as planned.

Training sessions participants reported these as useful, succinct, and professionally delivered. Participants particularly appreciated the one-on-one nature of the Portal User Training, which was interactive (a *“learn by doing”* approach; E_PSYCH_S08P04T1), tailored to their role and allowed them to ask questions.

Fidelity in delivery of education relies on capacity of services to facilitate staff attendance, which is dependent on the needs of the clinical area on a given day and time. Flexibility and adaptability in meeting needs of part-time and shift-based workforce, and staff turnover is also required. Participants commented that education needed to accommodate this to optimise effectiveness. Advance scheduling, flexible and adaptable delivery and keeping sessions short helped to maximise attendance as it enabled staff to fit this in alongside their clinical loads.

*“we’ve got a very small permanent staff base at the moment so [training] will need to be rolled out again as we recruit… repeating them keeping in mind that the, kind of, issues associated with a rotating roster and actually capturing people.”* (E_NURS_S09P02T0).

At T1 and T2, some participants commented that they received additional training from the ADAPT Team during the implementation period (e.g., staff new to the service, returned from secondment/ leave, or missed initial training), which was viewed positively.

*“nothing was too much trouble. Every person was trained…any new staff that we had new training needs… they did that, and were really very flexible, very accommodating… I guess without that…everyone would have gone, no, this is too hard.”* (E_NURS_S02P08T2)

#### Impact of education on preparedness

Most staff felt they had been provided with adequate information about the CP and Portal to start implementing the ADAPT CP at their service.

At T0, participants felt they needed to use the ADAPT CP and Portal ‘in practice’ to solidify the knowledge and skills learnt during training. Whilst they thought implementation would initially be challenging, they expressed faith that it would get easier over time and with practice, and that problems which arose would be overcome.

*“there’s probably going to be things that we need to iron out and problem solve as they crop up… we haven’t got it perfect… but I think we’re prepared enough to start actioning it.”* (E_PSYCH_S07P02T0)

*“once I start playing around with the portal…registering patients… really getting stuck into it, it’s going to be a lot easier”* (C_ADMIN_S01P05T0)

Training was perceived to be most effective when delivered close to staff enactment of the CP and Portal. Time-lags between training and first use, or intermittent use of the CP and Portal, were barriers to preparedness as it meant information was less likely to be retained and staff had to *“re-learn things again”* (C_NURS_S01P01T2). Refresher training just prior to Go-Live, or throughout the 12-month implementation period when staff were enacting the CP, were suggested.

Using the ADAPT CP and Portal ‘in practice’, contacting the ADAPT Support Service, and referring to the User Guides and Quick Guides helped to overcome issues and increased preparedness.

### Fidelity to academic detailing and support

Champions were provided with a written (de-identified) report summarising amongst other issues that were explored during the staff interviews, staff acceptability of the ADAPT CP implementation (T0, T1, T2). Reports were sent to the ADAPT Champion at all services, as planned, after completion of staff interviews at each timepoint.

#### Engagement meetings

All services participated in Engagement Meetings to prepare for the implementation of the ADAPT CP, and challenges and benefits of convening a local Lead team regularly with the ADAPT team were evident. Across the results, we noted a lack of consistency in using the Engagement Meetings strategy (6-8 planned meetings spaced about 2 weeks apart). Reasons for this included non-availability of Lead Team staff, and service desire to move efficiently through decision-making. For example, at one service, delays in forming a Lead Team and securing multidisciplinary attendance stalled decision-making and led to a protracted Engagement Phase (*n*=12 meetings across 41 weeks) (Table 5). Meetings were in-person (*n*=55, 69%) or via teleconference (*n*=25, 31%) and involved 402 attendees (*Mean*=34 per service), the majority (*n*=370, 92%) lead team members. The time from first Engagement Meeting to CRCT implementation/Go-Live was, on average, 25 weeks (range 10-41 weeks).

**Table 5:** Number, duration and attendances to the engagement meetings

**SID**	**Engagement Meetings**		
	**Total Number of Meetings (n)**	**Total Meeting Duration (mins)**	**Total Attendances (n)**
1	7	391	30
2	4^a^	175	24
3	7	315	35
4	7	450	35
5	7	420	24
6	12	710	64
7	6	195	35
8	7	295	27
9	5^b^	235	25
10	7	380	43
11	4^c^	240	16
12	7	455	44
**Total**	**80**	**4261** (71h1m)	**402**
**Average (per site)**	**6.67**	**355.08** (5h55m), (53 m per meeting)	**33.50**

^a^Site 2 resolved Engagement Meeting Agendas 5,6,7 via email at site request.

^b^Site 9 combined Engagement Meeting Agendas 4/5, 6/7

^c^Site 11 combined Engagement Meeting Agendas 1/2, 4/5 and 6/7 at site request

On a more positive note, those who attended appreciated their structured nature of having clear agenda facilitated focused decision-making, were succinct, and had a clear purpose.

*“they were really quick and they ran on-time… straight to the point… there was a purpose behind them and they answered the questions, and yeah, the agenda was set up really well.”* (C_PSYCH_S03P03T0)

Having time between meetings enabled tailoring decisions made during meetings to be discussed outside meetings and later finalised or revised. However, some participants noted too many meetings, or too much time spent during meetings revising tailoring decisions made previously, which ensured preparedness but was perceived as *“overkill”*. These participants suggested that meetings be condensed, for example by minimising the time spent reviewing tailoring decisions or having fewer meetings that lasted longer.

At one service, adaptations to meeting mode (i.e., held via email) at the service’s request, which reduced burden and enabled them to fit within staff workload/flow.

*“[ADAPT Program Manager] tailored it to suit us a little better by disbanding the actual telehealth meetings and actually doing a lot of it via email, which was really good… [engagement meetings] were only an hour but that was a big, big drain on trying to do your work and getting the meeting and dedicating that time. A lot of information very quickly, but at least it was backed up with emails and sort of discussion that way.”* (E_NURS_S02P05T0)

#### Impact of engagement meetings on preparedness and ownership

Having a series of meetings prior to Go-live increased staff awareness of ADAPT and ensured staff from different disciplines were *“on the same page”* (E_PSYCH_S07P02T0) about how the CP would be enacted at their service and what was required of them.

*“we just managed to build that momentum through those meetings so that the key people, the psychologists, the clinical trial staff, senior nursing staff in the clinic and doctors all had enough awareness, that meant when it went live, it was fresh, and they were really able to capture people.”* (E_MED_S06P02T0)

However, one participant commented that staff roles/responsibilities needed to be more clearly specified from the earliest engagement meetings to maximise engagement.

*“if you were slightly ignorant of what’s going on and not quite clear about what you are being asked to do, there’s sort of a sense of look, I’m pretty busy anyway, and if this means extra work for me, and, you know, my team, well, then maybe I won’t do it.”* (E_MED_S06P02T0)

Participants described working collaboratively with the ADAPT team during the engagement meetings to tailor the CP to their site. The ability for staff to have input into their ADAPT roles also promoted a sense of ownership and ensured roles fit their skills, experience and capacity.

In contrast, nursing staff at one site felt they had limited input during the meetings and were concerned that their ADAPT roles had the potential to exceed their scope of clinical practice in delivering psychosocial care.

*“I know in nursing we do deal with… mental health and that, but…in terms of triaging it feels like it is, you know, we’re not trained psychologists and even though a patient may be at a level two in their survey it feels like it could lead to a lot more.”* (E_NURS_S04P03T0)

#### Monthly meetings (enhanced strategy arm services)

Monthly meetings with the eight enhanced strategy arm services were scheduled in advance (*n*=96); timing and mode of meetings was adapted to maximise lead team attendance. Eighty-four monthly meetings of on average 34 minutes, were held (on average 10 meetings per service) (Table 6). Meetings were held in-person (*n*=41, 49%), online (*n*=35, 42%) or via teleconference (*n*=8, 10%). Reasons for discrepancies in the number of meetings delivered (*n*=84 compared to 96 planned) included cancellation upon service request and lead team unavailability (e.g., leave, holiday period).

**Table 6:** Number, duration and attendances to the monthly meetings

**SID**	**Monthly Meetings**		
	**Total Number of Meetings (n)**	**Total Meeting Duration (mins)**	**Total Attendances (n)**
2	10	331	59
4	10	400	54
5	11	310	33
6	11	545	50
7	10	310	33
8	12	330	42
9	10	320	56
10	10	300	39
**Total**	**84**	**2846** (47h26m)	**366**
**Average (per site)**	**10.50**	**355.75** (5h56m), (34m per meeting)	**45.75**

Participants from Enhanced services found the monthly meetings valuable; they gave staff a chance to review ADAPT progress at their site, identify emergent implementation barriers and brainstorm possible solutions. Discussing site progress throughout the implementation period also helped to maintain staff engagement and facilitated team cohesion.

*“keeping people updated it creates a bit of buy in as well so people remain interested in, enthusiastic and involved”* (E_AH_S06P03T2)

*“we always use that meeting as a forum for bring up problems and getting solutions of those problems” (E_NURS_S02P08T1)*

Staff were positive about the ADAPT Team facilitators, who drew on previous implementation experience to help services come up with solutions to identified barriers.

*“[the ADAPT Team] tried to really brainstorm, specifically what was going on at our local site. They didn’t pass judgement. They weren’t saying you’ve got to do better or anything like that… trying to facilitate us coming up with the answers which I thought was excellent.”* (E_AH_S10P07T2)

However, some staff commented that meetings were difficult to attend for example due to competing clinical demands, shift work, or that there was inadequate representation (particularly of senior management staff) to optimise effectiveness.

*“it’s all very well that we’d come up with any ideas of what can happen, what could happen… but unless there’s higher management there to action it … it makes it really difficult” (E_AH_S06P03T2)*

Participants from Core services reported having informal conversations about ADAPT with other staff, adding short discussions about ADAPT to existing meetings (e.g. team or steering committee meetings) or trouble-shooting issues individually as they arose.

A few Core participants commented that regular meetings throughout implementation would have helped to identify and address emergent implementation barriers and maintain staff engagement – which were otherwise difficult to raise.

*“[meetings would] open up a dialogue about what’s working, what’s not working, what we can do differently, what we can do better”* (C_PSYCH_S11P01T1)

### Fidelity to reports

Twenty-two staff across all services generated at least one report in the ADAPT Portal (*Mean=2* staff per service, range = 0-6 staff); most were local Champions or Portal Site Administrators (*n*=13, 59%). Across all services 525 reports were generated, most frequently the Screening Report (*n*=190, 37%), which provided a summary of patient screening events in the reporting period and the Planned Notifications report (*n*=108, 21%), which showed upcoming notifications to be sent to cancer service staff.

Content of monthly reports were adapted upon service requests. Few (*n*=3) enhanced strategy arm sites requested additional information for inclusion in these reports, to enable them to focus on specific data of interest, such as patient registrations by tumour streams or clinical departments. The monthly reports enabled staff to reflect on their service’s progress and identify and resolve emergent issues. They also helped to maintain staff engagement by *“keeping the program on people’s radar”* (C_MED_S11P05T1). At one site, reports showed that no referrals to psychosocial support had been made despite a number of patients screening high prompting staff about the lack of psychosocial supports available, and subsequently new referral pathways were identified. Several staff commented that the Portal-generated patient reports helped them to identify and understand patients’ issues, which facilitated triage conversations and treatment.

*“because some of the people that were screening, I was already seeing or…were getting referrals for from other sources. So, the information on their screening…was useful for me when I was seeing them.”* (E_PSYCH_S02P01T1)

### Fidelity of technical support

There were approximately 485 contacts between service staff, patients and the ADAPT Team during the engagement and implementation periods. Approximately 37% of contacts (~ 179 contacts) related to registering patient or staff on the ADAPT Portal and log-in issues followed by technical issues related to online screening (approximately 99 contacts).

Most participants reported being aware of the ADAPT Support Service and an ability to access this at any time. Some participants reported having used the service, whilst for others there had been no need. Participants who accessed the service were positive about this strategy and reported it was easy to access and that the ADAPT Team were approachable, *“readily available”* (E_PSYCH_S10P04T0) and responded quickly. The ADAPT Support Service helped participants to implement the CP and troubleshoot issues as they arose.

*“If I had to email about something or I’d queried something, it’s been immediate, they’ve come back… they’ve been supportive and they’re wanting this to work and wanting to work with us. So, I’ve felt that has really set the standard for me that, you know, this is a relationship and I’m sure that we can make this work*.” (C_PSYCH_S01P03T0)

## Discussion

In this paper, we have reported fidelity to and the acceptability of six categories of implementation strategies, as illustrated by staff perceptions during a 12-month supported implementation of the ADAPT Clinical Pathway. We have also reported any subsequent changes/adaptions made to the implementation strategies and reasons for these differences. All six categories of implementation strategies were delivered and were acceptable to staff. Although most strategies were delivered as planned, there were variations or modifications in terms of the content, frequency and duration of some of the strategies delivered to services, meaning that adaptation was a necessary and potentially, even desirable, feature of implementation strategies.

As noted in Carroll et al., (2007)’s framework, quality of delivery (i.e., delivery of intervention in an appropriate way to achieve what was intended) and participant responsiveness may impact on the coverage, frequency or duration of implementation fidelity [24]. In our study, securing staff engagement at some services was easy, and time was made for delivery of ADAPT implementation strategies; at other services, this was difficult, and scheduling of Roadshow, training sessions and meetings had to “fit-in” within existing meetings, be condensed or delivered via email, potentially affecting fidelity to the strategy and hence implementation success. Furthermore, training gave staff an insight into the ADAPT CP and Portal and how they would work in practice, which catalysed a shift in staff engagement at some services.

Adaptation of implementation strategies and flexibility to accommodate service needs appeared to have a positive impact on implementation fidelity. Most staff felt the Roadshows, training sessions and meetings were important in facilitating staff preparedness, ownership, engagement and to trouble-shoot emerging implementation barriers. However, busyness of service staff, staff turnover and nature of staff employment (such as part-time or shift work patterns) affected engagement. Awareness of implementation context, modification and flexibility (content, duration, frequency and mode of delivery) could act as facilitators to engagement and implementation fidelity.

Our results also highlighted the importance of senior management to endorse implementation, promote staff engagement and optimise the effectiveness of the local Champion strategy. Similar to existing research [26, 27], our results demonstrate the need to consider, not only the role of the Champion, but also attributes that may influence a Champion’s ability to drive service change. Authority or support from upper management and designated time to dedicate to the role may maximise the effectiveness of Champions as an implementation strategy.

Ongoing implementation support is crucial, especially in initial phases of service change or adoption where logistics of implementation are fine-tuned to optimise success. Our research team provided additional training to staff throughout implementation upon site request. This adaptation was acceptable to staff and facilitated awareness and engagement with implementation. Furthermore, most staff at services randomised to the enhanced strategy arm, believed the ADAPT-facilitated monthly meetings contributed to implementation success.

There are limitations to this paper. Although we were able to collect quantitative data on staff engagement with the newsletters, this was not possible for most of the awareness campaign strategies (e.g., number of staff or patients that viewed posters, or number of staff that opened the emails). This limited our understanding of the extent to which staff engaged with these strategies and thus the extent to which these strategies may have been adapted or modified and any impact that this had on adherence to the ADAPT CP. We also had large variations in the duration of the Portal Overview training as some services only received an abbreviated five-minute presentation at the end of the Clinical Pathway training due to lack of additional time. Whilst there was low fidelity to the Portal overview training which provided an overview of the ADAPT Portal and its functionalities, the tailored Portal User training and/or Super User training were provided to all staff who implemented or used the ADAPT Portal*.* Future research should also include quantitative measures of fidelity to determine impact of implementation fidelity on intervention success.

## Conclusion

This current study describes and documents the fidelity, engagement with and acceptability of six implementation strategies developed to assist with implementation of the ADAPT CP into routine cancer care. The findings add to the scarce literature detailing implementation strategies and how they are used and adapted in real-world trials. Clear documentation of fidelity to and understanding of the acceptability of strategies will inform the appropriate selection and design of implementation strategies in future studies and helps to support implementation of interventions in healthcare settings.

### Supplementary Information

**Additional file 1.** CONSORT Flow Diagram for ADAPT cluster RCT.

**Additional file 2.** Additional Quotes on staff perceptions of implementation strategies. Table providing additional quotes on staff perceptions of implementation strategies.

## Data Availability

The datasets used in this study are available from the corresponding author on reasonable request.

## Abbreviations

ADAPT CP: Clinical Pathway for identification and management of anxiety and depression in adult cancer patients.
CRCT: Cluster Randomised Controlled Trial
