# Supplementary material for: Fidelity and acceptability of implementation strategies developed for adherence to a clinical pathway for screening, assessment and management of anxiety and depression in adults with cancer
Source: Arch Public Health. 2024 May 6;82:65. doi: 10.1186/s13690-024-01293-6 (PMC11071180; doi:10.1186/s13690-024-01293-6)
Supplement: Supplementary file 1 — Additional file 1. CONSORT Flow Diagram for ADAPT cluster RCT. [file 13690_2024_1293_MOESM1_ESM.docx]

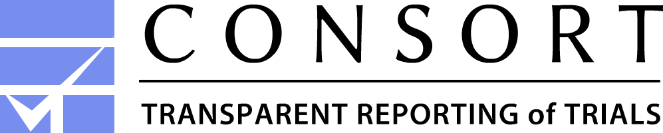


**CONSORT 2010 Flow Diagram**

Invited expression of interest (n=25 services)

## Enrolment

Excluded (n=13 services)

♦  Declined to participate (n=13)

Randomised (n=12 services)

## Analysis

**‘Enhanced’ cluster** (n=8 services)

♦ Staff completed T1 surveys (n=35)

♦ Staff consented and completed T1 interviews (n=59)

**‘Core’ cluster** (n=4 services)

♦ Staff completed T1 surveys (n=23)

♦ Staff consented and completed T1 interviews (n=30)

## Time 1

**Allocated to ‘Enhanced’ cluster** (n=8 services)

♦ Staff completed T0 surveys (n=76)

♦ Staff consented and completed T0 interviews (n=61)

**Allocated to ‘Core’ cluster** (n=4 services)

♦ Staff completed T0 surveys (n=30)

♦ Staff consented and completed T0 interviews (n=27)

**‘Core’ cluster** (n=4 services)

♦ Staff completed T2 surveys (n=26)

♦ Staff consented and completed T2 interviews (n=29)

**‘Enhanced’ cluster** (n=8 services)

♦ Staff completed T2 surveys (n=31)

♦ Staff consented and completed T2 interviews (n=47)

♦ Analysed (n=4 services)

♦ Analysed (n=79 staff surveys)

♦ Analysed (n=86 staff interviews)

♦ Excluded from analysis (n=0 services)

♦ Excluded (n=0 staff from surveys & interviews)

♦ Analysed (n=8 services)

♦ Analysed (n=142 staff surveys)

♦ Analysed (n=167 staff interviews)

♦ Excluded from analysis (n=0 services)

♦ Excluded (n=0 staff from surveys & interviews)

## Allocation

## Time 2
