# Supplementary material for: Fidelity and acceptability of implementation strategies developed for adherence to a clinical pathway for screening, assessment and management of anxiety and depression in adults with cancer
Source: Arch Public Health. 2024 May 6;82:65. doi: 10.1186/s13690-024-01293-6 (PMC11071180; doi:10.1186/s13690-024-01293-6)
Supplement: Supplementary file 2 — Additional file 2. Additional Quotes on staff perceptions of implementation strategies. Table providing additional quotes on staff perceptions of implementation strategies. [file 13690_2024_1293_MOESM2_ESM.docx]

Additional File 2. Additional Quotes on staff perceptions of implementation strategies.

| **1. Awareness Campaigns** | *“I think they were helpful. Just to kind of get the ball rolling, get the awareness out there.”* (C_NURS_S12P01T2)  *“with the posters and the emails, and the face-to-face sessions, it was really… capturing everybody’s attention… so when it went live… everybody was on board with it… we…registered lots of patients in the first few weeks which was really good.”* (E_AH_S06P03T2) |
| --- | --- |
| - 1. *Roadshows* | *“an in depth understanding of what was going to happen, why it was needed… and working about – how it would look like and how it would be run.”* (C_ADMIN_S01P08T2)  *“I think [the Roadshow was] when we, sort of, got a lot more people on board”* (E_ADMIN_S02P05T2)  *“[staff have] got other things happening, like, now and next week… I think it’s more beneficial to do…the training and the awareness nearer to the time, so it can be, this is happening, and then you just roll into it happens”* (E_AH_S06P03T0)  *“I think maybe if there was an extra, just even like a two minute come and introduce, we met last year or how many months ago. ADAPT is about to go-live. This is what is expected from you.”* (E_NURS_S07P04T0) |
| *1.2 Posters and Emails, Newsletters* | *“I think they just probably gave people exposure… probably the lead team had a good sense of what was going on but perhaps other people in the service didn’t. So I think that was just…kind of raising awareness stuff.”* (C_PSYCH_S11P01T0)  *“sometimes staff read the e-mails, sometimes they see too many e-mails they're just not interested.”* (C_NURS_S12P05T1)  *“That’s probably the best way of doing it, really. People actually sort of sitting there and listening to someone enthusiastically talking about it, because you know what it’s like in a hospital. You’ve got posters everywhere for everything and they kind of vie for attention, really.”* (E_NURS_S10P08T1) |
| **2. Perceptions of Champions as an Implementation Strategy** | *“I think it was probably good, so that there's someone on the team who, you know, carries the self-responsibility, and that responsibility doesn’t get diffused.”* (E_PSYCH_S07P01T1)  *“I think [having a champion] makes it easier for us ‘cause [champion]’s been involved the whole way so… as the implementation occurs and the modifications arrive I think [champion]’ll be able to hone that for us and then she’ll skill us up as the need arises.”* (C_MED_S11P04T0)  *“you knew there was like a designated person to go to if you had any questions.”* (E_NURS_S07P09T1) |
| *2.1 Attributes of a Successful Champion*  *2.1.1 Role* | “*my colleague, she is the champion…that’s definitely helpful because, ah, if I have had questions or will have questions, I feel confident going to her and her helping me work it out*.” (E_PSYCH_S08P06T0)  *“[clinical trials staff, champion] did a great job… but I wonder whether it needs to be clinical staff. I mean, down the track I guess if you keep doing this sort of service it needs to be clinical more so than, um… trial staff.”* (C_AH_S03P01T2) |
| *2.1.2 Authority* | *“it’s hard. Because you can have the best champions operationally within the group um, and unless you get that conformity – from the people that matter… you miss out on it working.”* (E_ADMIN_S08P05T2) |
| *2.1.3 Workload Capacity* | *“I think it’s a good idea, but I think it just depends on who the champions are and how proactive they’re going to be and how much time they’ve got to… promote the project”* (E_NURS_S10P02T0)  *“the champions are good, however, the champion we had at the time… it just was too hard for her, because she had her priorities on the ward, her patients, so she didn’t really have the time”* (E_NURS_S07P04T2)  *“it’s probably a helpful type of approach to take with rolling out something new if you have funding. Because everybody else seems they’re busy in their current role. To take on something new, becomes difficult and you can’t always put the correct amount of energy or time into it and so things tend to drop lower on the priority scale. Whereas when you have someone who’s … role is dedicated to that for so many hours a week. I think that works quite well.”* (C_AH_S12P06T1)  *“we haven’t had that much exposure on the ward, because [nursing staff, lead team] do it for us.”* (E_NURS_S07P09T1) |
| **3. Perceptions of Education** | *“it's explained to you and shown on the screen…then you can have back and forwards conversation of … how to work it and so forth.”* (C_AH_S03P01T2)  *“when you actually got to look at [the ADAPT Portal] and see what’s going to happen, one-on-one, and it was explained to you, it made it… a lot more clearer and lot more sense.”* (C_NURS_S03P08T0)  *“It wasn’t until [ADAPT Program Staff] came up for a couple of meetings and went through with her presentation that you actually saw a big change with people… it sort of allayed their fears that it’s not going to be, you know, really cumbersome and bogged down… once [ADAPT Program Staff] showed everybody how the – what the portal looked like, what the reasoning was, and a bit of an overview of where it all went, it became a more sort of unified approach for the rest of the unit.”* (E_ADMIN_S02P05T0) |
| *3.1 Impact on Preparedness* | *“I feel confident and I feel that it was explained well”* (E_NURS_S04P03T0)  *“we’ve all had…good access to training um, and education about the, how to use the portal and the pathways and… I can feel confident using it within the psychosocial team and I’m happy to help other people if they don’t feel confident.”* (E_PSYCH_S04P08T2)  *“practice makes perfect, so if we get through a few… we’ll be fine… we’ll work it out.”* (E_PSYCH_S10P01T0)  *“It was a long time between the training and the go-live that I ever got a referral. So that impacted me ‘cause I then had to go back, I had to reread, I had to rethink, I had to get back in, I had to look back at the system ‘cause it’s not a system I was using.”* (C_PSYCH_S01P03T1)  *“making sure that the training is as close to the, um, go-live date as possible.”* (E_PSYCH_S09P03T0)  *“with so many patients on it, it’s made it a lot easier and after you’ve used it for a little while, it’s getting…easier to do it, it’s just more about repetition”* (C_NURS_S01P04T1)  *“[the user guide] is really helpful because… it’s a guideline of whenever we forget something.”* (C_ADMIN_S01P02T2) |
| **4. Academic Detailing and Support**  *Perceptions of Engagement Meeting Delivery, Content and Frequency* | *“it was very clear at the end, who was doing what in preparation for the next meeting*.” (E_NURS_S05P08T0)  *“We’d run through the workflow and then we’d come up with who’s going to be looking into what… and then we’d come back with the answers for the next meeting and… move it along. I think because there was lots of different departments involved… we needed to have those meetings.*” (E_AH_S06P03T0)  *“I think if we’d probably just nutted it out in a couple meetings it probably would have been more effective”* (E_NURS_S07P06T0)  *“I think there was too many, too little information in each meeting. I would have much rather… longer and just, you know, get it all done in a couple of meetings.”* (NURS_ S07P04T0) |
| *4.1 Impact on Preparedness and Ownership* | *“It gave all staff a chance to ask questions and get a little bit of clarity over what’s happening and why.”* (E_ADMIN_S05P03T0)  *“if we hadn’t had the meetings and it was like, you’re doing this, I probably wouldn’t have been as invested because I wouldn’t have understood why am I doing this or what – what’s all this about… I think that… helped implement it, yeah.”* (E_AH_S06P03T1)  *“They listened to all of the ideas and we worked through it all together… lots of input… for me, for the registering of the patients because that’s my part of the role, but I know that other people had a lot of say in their parts of the role as well.*” (E_AH_S06P03T0) |
| - 1. **Perceptions of Monthly Meetings** | *“It felt like you were talking to people who really got what you were dealing with”* (E_PSYCH_S06P04T2)  *“clinical demand is growing... on top of that, we had the additional role of trying to fit the ADAPT Programming there. But… it was unavoidable really. We had to have those meeting to coordinate things better and in many ways, it saved time as well”* (E_PSYCH_S06P01T2)  *“We haven’t even met as a group to say, what are you experiencing? It’s just conversations when we see things happen”* (C_PSYCH_S01P03T1)  *“It does get caught up at the supportive care meeting… which is part of the hospital team meeting. But… it’s touched on quite lightly and doesn’t really get into the nitty gritty of it.”* (C_ADMIN_S01P08T1) |
| **5. Perceptions of ADAPT Reports** | *“to see how we’re doing and where we’re actually falling down and what we need to focus on really.”* (E_PSYCH_S10P01T1)  *“[receiving reports] was useful at a macro level. Just in terms of…how we were going in terms of screening and noting when the screening really dropped off and talking to the team, and trying to explore the barriers and trying to improve that.”* (C_MED_S11P05T2)  *“So that feedback was excellent. Because, I think, sometimes… there did seem to be this barrier to patients being referred on.”* (C_AH_S03P02T1)  *“so that just aided in having a chat with the patient…reminding me what things were issues for them and just having a chat about those...”* (C_NURS_S03P05T2) |
| **6. Technical Support** | *“I’ve always had very prompt responses. So I felt like that that was always very accessible support.”* (E_PSYCH_S08P06T1)  *“I didn’t have time to become an expert in everything ADAPT portal… so when I did come across an issue that needed problem solving…It was great to have that service there to, contact and they were always very prompt and very effective at responding to those.”*(E_PSYCH_S09P03T2) |
